# A Comprehensive Approach to Rehabilitation Interventions in Patients with Angelman Syndrome: A Systematic Review of the Literature

**DOI:** 10.3390/neurolint13030036

**Published:** 2021-07-28

**Authors:** Maddalena Sommese, Bruno Corrado

**Affiliations:** Department of Public Health, University of Naples Federico II, 80131 Naples, Italy; maddalena.sommese@gmail.com

**Keywords:** Angelman syndrome, happy puppet syndrome, Puppet Children, disability, rehabilitation, physical and rehabilitation medicine, physical therapy modalities

## Abstract

Angelman syndrome is a rare genetic disease affecting the central nervous system and neurobehavioral development causing severe mental, linguistic, and physical disabilities. The purpose of this review was to analyze the most recent evidence regarding the rehabilitation of subjects affected by this syndrome. The review was carried out in accordance with the preferred reporting items for systematic reviews and meta-analyses. A total of 3661 studies were identified in the databases. Once the inclusion/exclusion criteria were applied, 15 studies were considered for the paper’s preparation. The level of evidence of the studies was established according to the criteria of the Oxford Center for Evidence-Based Medicine—Levels of Evidence. From the selected studies, five rehabilitative approaches emerged: physiotherapy, applied behavioral analysis, toilet training, microswitch-cluster technology, and augmentative and alternative communication. Although the studies did not have a high level of evidence, the reported results appear to be encouraging and pave the way for further studies. It seems that individualized and multidisciplinary rehabilitation interventions help to improve patients’ autonomy and quality of life. In some studies, the caregivers’ role was fundamental to identify preferences and long-term improvements. Further studies on larger populations and with better methodological quality are needed to confirm the results.

## 1. Introduction

Angelman syndrome (AS) is a neurodevelopmental disorder characterized by severe intellectual and motor delay, marked speech impairment, peculiar facial expressions, and an autistic-like behavioral pattern.

Harry Angelman, an English pediatrician, first described this condition in 1965 when he reported three children that he referred to as “Puppet Children” because of their unusual arm positions and jerky movements [1]. The incidence of AS is unknown [2]. It has a prevalence between 1/10,000 and 1/20,000 [3,4]. AS has been detected all over the world and across all races [2].

In about 80% of patients, AS is due to the lack of function of the ubiquitin-protein ligase E3A (UBIE3A) gene, mapping to chromosome 15q12-q13 [2,5]. Expression studies in embryos of mice have shown a paternal imprinting of the UBE3A gene restricted to the developing brain, particularly involving Purkinje cells, hippocampal neurons, and mitral cells of the olfactory bulb [6].

The lack of function of the UBE3A gene may be due to: (1) the deletion of the 15q11-q13 region of maternal origin (70%); (2) chromosome 15 paternal uniparental disomy (UPD)—in this case, both copies of chromosome 15 are paternal in origin (3%); (3) imprinting center (IC) mutations (1%); and (4) intragenic mutations of the maternal copy of the UBE3A gene (6%).

Consensus criteria for the clinical diagnosis of AS have been developed in conjunction with the Scientific Advisory Committee of the Angelman Syndrome Foundation [7,8]. Newborns typically have a normal phenotype. Developmental delays are first noted at around the age of six months. However, the unique clinical features of AS do not manifest until after one year of age, and it takes several years before the correct clinical diagnosis becomes obvious. The diagnosis is usually first suspected on the basis of the behavioral phenotype, particularly combinations of movement disorder, absent speech, and happy demeanor. Although some individuals with AS may have apparent mild craniofacial dimorphism, the diagnosis is rarely suspected based on these findings [8]. 

Though most individuals lack speech entirely, some who are mildly affected can acquire a few words. Receptive language is less impaired. Seizures occur in >80% of patients, and their onset is usually before the age of three years. Movement disorders include tremors, jerkiness, and ataxia. Voluntary movements are often irregular, varying from slight jerkiness to uncoordinated coarse movements that prevent walking, feeding, and reaching for objects. The characteristic behaviors of AS include mouthing of objects, happy demeanor with easily provoked laughter, attraction to water, hyperactivity, short attention span, and decreased sleeping [4,8,9,10]. In addition, there are psychomotor developmental delays (autonomous walking is usually not acquired before two years of age), and severe mental retardation (IQ < 50, or subjects not testable). Such features of AS can be seen in other neurodevelopment disorders too, leading to a broad differential diagnosis [11].

To date, there is no cure for disorders such as AS because there are still no definitive ways of repairing chromosomal defects or of restoring function to mutated genes. At present, optimal care management and rehabilitation are the only options for improving the health-related quality of life in children affected by neuromuscular disorders [12,13,14,15,16,17,18,19,20,21]. In such conditions, the goal of rehabilitation is to achieve and maintain optimum functioning in interaction with environments, following the International Classification of Functioning (ICF) approach to disability, which understands functioning and disability as a dynamic interaction between the health condition and contextual factors, both personal and environmental [22,23,24].

Treatment and management of AS focus on managing the physical and neurologic problems of the patient and providing appropriate educational support [25]. Management of AS is symptomatic, meaning that the therapy itself is usually aimed at reducing the signs and symptoms of the syndrome for the comfort and wellbeing of the patient, but does not address the basic cause of the disease itself [26].

Because AS is a multisystem disorder, patients with AS require multidisciplinary interventions throughout their lives.

The general health of patients with AS is fairly good and life-span may be near normal if the patient is healthy and does not have sever epilepsy or cardiorespiratory problems, which may complicate severe scoliosis [27].

Particular problems which have arisen in adults are a tendency to obesity, worsening of scoliosis, increased incidence of joint contractures, and esophageal reflux [28,29].

## 2. Materials and Methods

This review was carried out in accordance with the preferred reporting items for systematic reviews and meta-analyses (PRISMA) [30].

### 2.1. Study Eligibility Criteria and Report Eligibility Criteria

The following types of paper were excluded from the study: guidelines, university theses, unpublished works in scientific journals, letters, and comments. Given the scarcity of scientific literature on the topic, studies were not limited to any particular design. The inclusion criteria applied were: (a) patients of any age with AS; (b) to which rehabilitative interventions (physiotherapy, speech therapy, occupational therapy) were applied; (c) no limits have been placed on the duration of the follow-up; and (d) completeness of data. The report’s inclusion criteria were: (a) written in English; and (b) published from January 2001 to December 2020.

### 2.2. Information Sources

With the aim of identifying relevant studies, a systematic review of the literature was performed using the following databases: PubMed (https://pubmed.ncbi.nlm.nih.gov/, accessed on 8 March 2021), Cochrane Library (https://www.cochranelibrary.com/, accessed on 8 March 2021), PeDro (https://pedro.org.au/, accessed on 8 March 2021), and Google Scholar (https://scholar.google.com/, accessed on 8 March 2021).

### 2.3. Search Strategy

The following keywords were used: ”Angelman syndrome”, “happy puppet syndrome”, “physical therapy”, “rehabilitation”, “occupational therapy”, and “AAC”, with Boolean operator “AND/OR”.

### 2.4. Study Selection

Initially, the titles and abstracts of the studies were evaluated, excluding those that did not meet the pre-established inclusion criteria. Studies that met the inclusion criteria were then reviewed in detail.

### 2.5. Data Collection Process

The data from the original articles were collected in data extraction tables specifying: general information on the study (first author and year of publication), study design, type of rehabilitation approach, number of participants, evaluation tools used, duration of follow-up, main results/findings, and level of evidence (grade of recommendation).

### 2.6. Level of Evidence Assessment Process

Levels of evidence were assessed using the Oxford Centre for Evidence-Based Medicine (OCEBM levels of evidence system) [31].

## 3. Results

From the preliminary database search 3661 studies were found: PubMed (*n* = 48), Cochrane Library (*n* = 0), PeDro (*n* = 0), and Google Scholar (*n* = 3613). First, studies with different design from that experimental or observational were excluded, such as book extracts, lectures, university theses, letters, and comments (*n* = 192). The titles and abstracts of the remaining 3469 studies were evaluated, with the exclusion of further studies that did not meet the pre-established inclusion criteria. In addition, studies reported more than once in databases were also deleted. This second phase lead to the exclusion of 3429 studies. Of the 40 remaining studies, the full-text version was evaluated. From these, 25 studies were excluded because they did not meet the inclusion criteria, while the remaining 15 were considered eligible. The selected results were classified as follow: 2 case reports; 2 case series; 1 ABB AB experimental sequence; 1 non-concurrent time series; 3 B-only design; 1 alternating treatment single-subject experimental design; 1 non-randomized, pre/post-test; 1 multiple-probe across participants and alternating-treatments design; and 1 pilot study.

From the detailed analysis of the articles, five rehabilitation approaches to patients with AS were identified: (1) physiotherapy; (2) applied behavior analysis (ABA); (3) occupational therapy: toilet training; (4) microswitch-cluster technology; and (5) augmentative and alternative communication (AAC).

**Figure 1:**
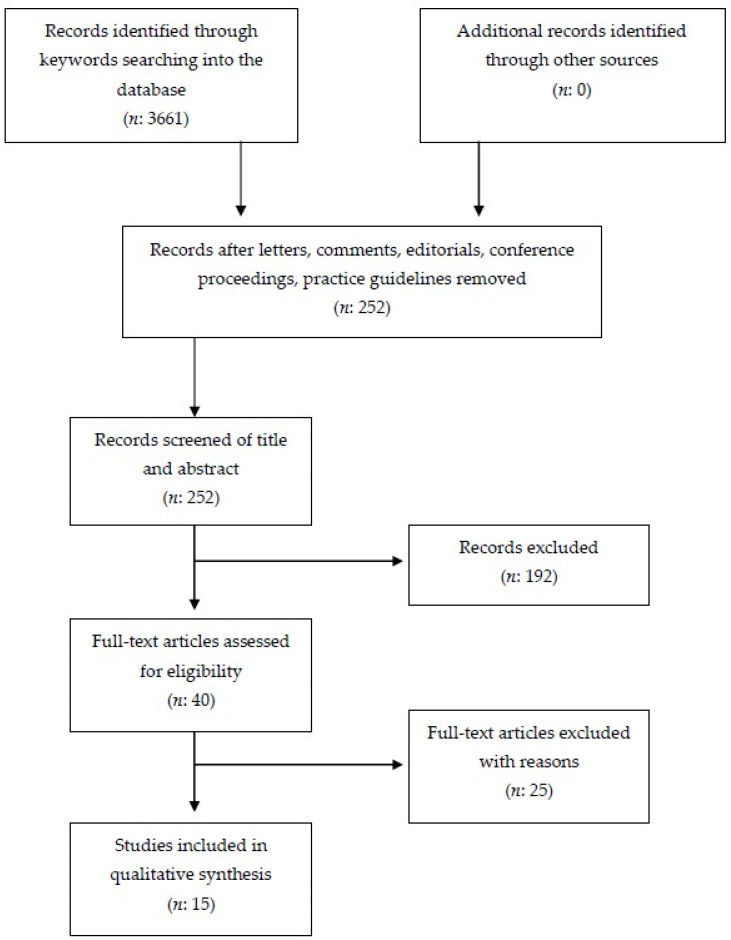
Preferred reporting items for systematic review and meta-analysis (PRISMA) flow chart concerning study retrieval and selection.

The PRISMA flow diagram used for study selection process is summarized in Figure 1. The details of the studies selected for the systematic review are listed in Table 1.

### 3.1. Physical Therapy

In the following two studies, physical therapy was applied to improve movements and balance deficits.

The results obtained with the neurodevelopmental treatment (NDT) in a single case of AS by Kara et al. suggested that a physiotherapy program started early and protracted over time could produce quantitative and qualitative changes in movements, improving the quality of life and autonomy of the patient [32].

Pessarelli Visicato et al. applied a physiotherapy program comprising balance exercises to a single case of AS [33]. The results suggested that although the intervention focused on static balance, it was also possible to improve dynamic balance at the same time. This improvement was relevant to the patient’s performance, since static and dynamic balance deficits in subjects with ataxia are correlated with a higher risk of falling. This data translates into a lower risk of falls, which is extremely important in order for the child to participate in socially relevant activities.

### 3.2. Applied Behavior Analysis (ABA)

The main goal of Summers’s study was to evaluate the impact of educational approaches based on ABA principles on neurological development in children with AS. A non-randomized, controlled pre-test-post-test design was used. Standardized measures of cognitive, adaptive, and language functioning were administered at the start and after one year. The author reported that children with a genetic mutation had a higher level of development so the program was more advanced than in children with genetic deletion. After one year of treatment there were improvements in the intervention group for receptive language and fine movements, although with a non-statistically significant result [34].

In another study by the same author, Summers, an ABA protocol was administered to 12 children to improve memory, imitative skills, and motor performance [35]. Imitation involves the ability to replicate observed behavior and is an important learning strategy through which children acquire and master new skills and behaviors. The administered protocol implied the copying of actions with the use of objects and was involved in the execution of life skills. These activities helped improve the quality of movements and activities of daily living in the 12 children with AS.

### 3.3. Toilet Training

Didden et al. first experimented the application of a modified toilet training protocol, “Azrin and Foxx” (1971), on six children with AS aged between 6 and 19 years. The results of the present study suggested that the modified toilet training program by Azrin and Foxx was effective in increasing the frequency of correct toileting. Follow-up data indicated that these effects were maintained after a period of 2.5 years. Parents and caregivers reported that some participants showed signs of self-initiated toileting. Furthermore, they reported that all participants showed less dependence in dressing [36].

Radstaake et al. used the response restriction (RR) method to improve continence. The study included six children aged between 6 and 25 who were able to sit for five min, who could walk independently, who had no seizure activity, and who were able to follow simple instructions. The questionnaires administered in the follow-up phase indicated that the positive results of the training were maintained in three participants. Furthermore, in one participant continence generalized to feces and nighttime continence decreased [37].

### 3.4. Microswitch-Cluster Technology

Stasolla et al., using an ABB AB experimental sequence, investigated the usefulness of a multiple microswitches-based program to promote object manipulation and to reduce tongue protrusion in seven children with AS. Long-term follow-up (i.e., 24 months) was performed. Secondly, 56 external evaluators were involved, equally divided into four groups (i.e., caregivers, physiotherapists, psychologists, and teachers), in a procedure of social validation of the results obtained. The microswitches adopted were a wobble microswitch for adaptive responding and an optic sensor for challenging behavior. The results showed that all participants successfully learned the functional use of the technology to improve object manipulation, decreased tongue protrusion, and positively participated in the sessions. All participants consolidated the learning process over two years when the follow-up was carried out. Social raters favorably evaluated the use of the technology and corroborated the social and clinical validity of the intervention [38].

### 3.5. Alternative and Augmentative Communication (AAC)

Roche et al. [47] carried out a first review of the literature on the use of AAC in people with AS.

The AAC modalities have been classified into three categories: (a) AAC modalities without the aid of technological aids; (b) AAC modalities with the use of technological aids; (c) multimodal AAC.

AAC without technological aids includes handwriting and natural gestures. AAC with technological aids include the use of image-based communication systems or electronic/computer-based speech generation devices. Studies using two or more modalities of AAC were classified as multimodal. Nine studies were examined: in three studies an approach known as enhanced natural gestures (ENGs) was used, in one study a picture exchange communication system (PECS) was used, while in the remaining five studies various types of AAC were utilized.

#### 3.5.1. Unaided AAC Modes

Calculator has worked on enhanced natural gestures (ENGs) in several studies. He first talked about this type of communication in a 2002 study [39]. With subsequent 2015 and 2016 studies, Calculator further experimented with this new type of communication in various life contexts [40,41]. ENGs, by definition, require a certain degree of education and motor skills.

#### 3.5.2. Aided AAC Modes

The picture exchange communication system (PECS) is a manual intervention protocol in which participants are taught to select and exchange illustrated cards. Image exchange is initially taught as a means of requesting access to desired objects. Radstaake et al. evaluated a PECS protocol within a study with an ABAB design. Participants were taught to make requests for objects or to attract attention by indicating an illustrated postcard containing the desired object or request to a listener who then honored their request. The results showed that the intervention resulted in an increase in targeted request responses and a reduction in the participants’ challenging/provocative behaviors to attract the attention [42]

#### 3.5.3. Multimodal AAC

The remaining five studies tested multimodal AAC interventions. Hyppa Martin et al. taught a boy with AS to request favorite objects by producing gestures and selecting graphic symbols. The relative successes of these two modalities were compared in an alternating treatment design. Although the child showed some improvement with each AAC mode, the correct response was consistently higher in the graphic symbol condition than in the gesture mode [43].

Radstaake et al. used symbols or a sound generation device to teach three children with AS to make requests for their own needs. The results indicated that all three children learned how to use their respective AAC modalities. Decreases in provocative behaviors were also noted [44].

Summers and Szatmari taught hand signs and an adapted version of the PECS protocol to three children with AS. The children were taught a variety of communication skills, such as waving, attracting attention, and requesting favorite items. One participant achieved the mastery criterion on four of the six targeted skills, while the other two participants made some gains but did not actually reach a language mastery stage [45].

In a 2012 study, Summers focused on teaching image exchange, the use of sound generation devices, and handwriting to three subjects with AS. A fourth participant was taught to express wants and needs through speech. Some positive effects were obtained, but there was no significant increase in overall expressive language scores for the children in the intervention group [34].

In the final multimodal study, van der Meer et al. taught a child with AS to request preferred items using sign language, image exchange, and a sound generation device with the aim of comparing the child’s preference. He learned to make requests in all three ways and seemed to show a preference for the sound generation device [46].

## 4. Discussion

Angelman syndrome is a rare neurodevelopmental disease, as such it affects multiple systems. The syndrome has a prevalence between 1/10,000 and 1/20,000, which allows it to be defined as a rare syndrome. There is no evidence that the life expectancy of children with this syndrome is lowered. However, AS is associated with a high prevalence of comorbidities. Early diagnosis and interventions to minimize secondary complications are crucial to maintain functioning and quality of life. An early and overall multidisciplinary approach is emphasized to maximize developmental potential for these individuals.

Performance of activities of daily living in patients affected by AS is variable. According to Guerrini et al., “Most individuals achieve continence by day, and some by night. Dressing skills are variable and are usually limited to items of clothing without buttons or zips. Most adults are able to eat using cutlery. Although no patient with AS is known to have been capable of living independently, they can learn to perform simple household tasks and are perhaps best suited to life in a community-care home” [2]. All AS patients require round-the-clock supervision because they have no sense of danger. It is important to encourage parents to take an active role in the management of their infant’s condition.

The only existing clinical guidelines regarding children with AS are those by Clayton-Smith et al. In such guidelines, a large space is devoted to the rehabilitation techniques analyzed in the present review. In addition, some alternative therapies are suggested. Many families report that alternative therapies for children with AS have a positive effect on wellbeing: hippotherapy, cranial osteopathy, aromatherapy, reflexology, hydrotherapy, music therapy, brushing, static cycling, and trikes. It must be noted that there is no scientific evidence to support the use of these therapies in AS [26].

What emerged from this systematic review is that different rehabilitation approaches can be used according to motor, functional, and communicative problems. A full range of educational training and enrichment programs should be available. Unstable or no ambulatory children may benefit from physical therapy, which can improve balance and movements if started early and protracted over time. Occupational therapy may help support personal autonomy, above all in toileting and incontinence. Speech therapy is essential and should focus on nonverbal methods of communication. Augmentative communication aids such as picture cards, ENGs, or communications boards should be used at the earliest appropriate time in order to encourage social interaction and avoid exclusion. In addition, the use of computerized devices, as MCT, may help improve the manipulation of objects and, at the same time, reduce provocative behaviors.

However, this review showed that available evidence for rehabilitation in AS is low. The level of evidence of the selected studies, in fact, ranged from 3 to 4 on the OCEBM scale. It was not possible to make a comparison between the results of the different studies and a meta-analysis. This is due to the heterogeneity of: (a) the times and modalities of the application of interventions, (b) the different ages of the subjects enrolled in the studies, (c) the different types of genetic damage and functional problems of the patients involved in the studies, and (d) the different types of assessment tool used.

Due to the heterogeneity of the studies and the population treated, and the low level of evidence of the studies, none of the proposed interventions can be considered better than others. Each of them can potentially be used to reach different goals which have as a common denominator an improvement in quality of life. In fact, the goal of the next frontier in healthcare for individuals affected by rare genetic conditions is to improve quality of life, not only by advancements in pharmacotherapy, but with interventions aimed at modifying psychosocial and contextual factors.

On May 2020, EURORDIS—Rare Diseases Europe announced preliminary global results from the first multi-country survey on how COVID-19 was affecting people living with a rare disease, finding that the pandemic had greatly hindered access to care. The COVID-19 pandemic has exacerbated the many challenges people living with a rare diseases already face and has created extra risks in their daily lives, with collateral consequences [48]. The interruption of rehabilitation programs has resulted in significant psychological and physical damage. The improvements obtained have been lost, with important development regressions. This demonstrates how important the continuity of rehabilitation interventions is throughout life [49,50].

This is the first systematic review concerning rehabilitation in AS. The present review was carried out following PRISMA reporting guidelines, with a clear, detailed, and reproducible methodology.

This review has several limitations: (1) the small number of studies selected for the final phase; (2) the fair methodology quality of the selected studies (e.g., small sample size, different assessments tools and intervention approaches used, short follow-up durations); and (3) the impossibility of carrying out a meta-analysis. It should be noted that it is common for all rare diseases that patients are dispersed, what makes difficult to carry out clinical trials with a significant number of samples.

Even if all studies included in this review demonstrated that rehabilitation in AS patients is necessary to improve their quality of life and autonomy, caution should be adopted in the interpretation of our findings.

## 5. Conclusions

What emerges from the present systematic review is that there are different types of rehabilitation interventions for motor, functional, and communicative problems in people with AS. Currently no single reviewed intervention can be recommended over another for the lack of cross-comparative studies, but there is some level of evidence for each individual therapy with regards to its specifically measured outcomes. Therefore, a personalized, multimodal rehabilitation program should be always suggested to people with AS, in order to improve function and quality of life.

Since the level of evidence in the scientific literature concerning the topic is currently low, future research should focus on carrying out studies with better methodology and higher levels of evidence.

## Figures and Tables

**Table 1:** Summary of the studies included in the review.

Title 1: Lead Author and Year of Publication	Study Design	Rehabilitation Approach	Number of Participants	Assessment Tool	Follow-Up Duration	Main Results/Findings	Level of Evidence (Grade of Recommendation)
Kara O.K. et al. (2010) [32]	Case report	Physiotherapy program based on NDT (Neurodevelopmental treatment)	1	GMFM, GMFCS, GMPM, BBS, and MAS	42 months	Increase in the total result of GMFM from 11.46% to 70.82%, increase of GMPM from 1.25% to 70.25%.	4 (C)
Pessarelli Visicato L., et al. (2013) [33]	Case report	Physiotherapy program	1	TUG, BBS (modified): the pediatric balance scale, biophotogrammetry	8 weeks	BBS: from 27 points to 37. 15 sec. TUG Test: from 15 sec to 12 sec. Biophotogrammetry: from 38 °C degrees to 13.78 °C degrees	4 (C)
Summers (2012) [34]	Non-randomized, pre/post-test	ABA	8	VABS, MSEL, and REEL-2	1 year	No significant differences between the groups but increased receptive language scores and hand manipulation for the intervention group.	3 (C)
Summers (2019) [35]	Pilot study	ABA	12	VABS—II, interview edition, survey form	3 months	Improvements in memory and motor performances.	4 (C)
Didden R. et al. (2001) [36]	Non-concurrent time series	Modified Azrin–Foxx toilet training procedure	6	Correct or incorrect voids	2.5 years	The percentage of correct urinations performed per day increased statistically significantly as opposed to the occurrence of incorrect ones that did not decrease statistically significantly.	4 (C)
Radstaake M. et al. (2014) [37]	Case series	Toilet training: response restriction (RR) approach	7	Number of correct voids and accidents	Different for each participant (18, 6–9, or 3 months)	Different results for each participant.	4 (C)
Stassolla F. et al. (2020) [38]	ABB AB experimental sequence	Microswitch-based program	7	VABS	2 years	Double goal achieved: increase in adaptive behavior and decrease in the number of problem behaviors.	3 (C)
Calculator (2002) [39]	B-only design	AAC: ENGs	9	ENG-ARF completed by the parents	18 weeks	With few exceptions, parents described this method as acceptable, effective, reasonable, and easy to teach others, with minor negative consequences and side effects.	4 (C)
Calculator and Diaz-Caneja Sela (2015) [40]	B-only design	AAC: ENGs	3	ENG-ARF completed by school staff	12 weeks	Two of the three students demonstrated particularly rapid and spontaneous uses of their ENGs.	4 (C)
Calculator (2016) [41]	quasi-experimental B-design	AAC: ENGs	18	Parents completed the ENG-ARF and the GAS	10 weeks	Children’s overall achievements acquiring ENGs generally met or exceeded program (and parent) expectations. Most parents reported little difficulty self-administering the ENG program with their children and regarded the program positively across multiple dimensions.	4 (C)
Radstaake et al. (2012) [42]	ABAB design	AAC: PECS	4	Vineland-Z	3–5 months	All children learned to independently exchange a referent picture or object, which resulted in a decrease in challenging behavior	4 (C)
Hyppa Martin et al. (2013) [43]	Alternating treatment single-subject experimental design	AAC: Vocal, gestural, and graphic communication modes	1	Narrative summery	N.S.	Vocalizations ranged from 0 to 40 per session	4 (C)
Radstaake et al. (2012) [44]	ABAB design	Functional communication training. Use of PECS and single-button SGD	3	Use of AAC to make requests	N.S.	Increases in AAC use and decreases in challenging behavior.	4 (C)
Summers and Szatmari (2009) [45]	Case series	Expressive sign language and PECS	3	MSEL, VABS, interview edition, REELS-2, module 1 from the ADO-G, Likert-scale surveys	12 months	One participant reached mastery on 4 of 6 target skills. Others showed improvement but did not reach mastery. Parents reported high levels of satisfaction	4 (C)
van der Meer et al. (2012) [46]	multiple-probe across participants and alternating-treatments design	PE, MS, and SGD	4 (only 1 with AS diagnosis)	Vineland-Z	2 weeks	During follow-up, the participant performance maintained at 100% correct for the SGD, but decreased to 40% and 0% correct for the PE and MS modes, respectively	4 (C)

GMFM: gross motor function measurement; GMFCS: gross motor function classification system; GMPM: gross motor performance measurement; BBS: Berg balance scale; MAS: modified Ashworth scale; TUG: time up and go test; VABS: Vineland adaptive behavioral scale; AAC: augmentative and alternative communication; ENGs: enhanced natural gestures; ENG-ARF: enhanced natural gestures-acceptability rating form; GAS: goal attainment scaling; PECS: picture exchange communication system; SGD: speech-generating device; MSEL: Mullen scales of early learning; REELS-2: receptive and expressive emergent language scale—second edition; ADOS-G: autism diagnostic observation schedule-generic; ABA: applied behavior analysis; PE: picture exchange; MS: manual sign.

## Data Availability

The data presented in this study are available on request from the corresponding author.
